# The Relationship between Self-Reported Executive Functioning and Risk-Taking Behavior in Urban Homeless Youth

**DOI:** 10.3390/bs8010006

**Published:** 2018-01-03

**Authors:** Joshua Piche, Jaeson Kaylegian, Dale Smith, Scott J. Hunter

**Affiliations:** 1 Pritzker School of Medicine, The University of Chicago, Chicago, IL 60637, USA; jpiche@uchicago.edu; 2 Department of Psychiatry and Behavioral Neuroscience, The University of Chicago, 5841 S. Maryland Ave., MC 3077, Chicago, IL 60637, USA; jkaylegian@gmail.com; 3 Department of Psychology, Olivet Nazarene University, Bourbonnais, IL 60914, USA; dsmith8@olivet.edu; 4 Department of Pediatrics, The University of Chicago, Chicago, IL 60637, USA

**Keywords:** homelessness, youth, executive functioning, risk behavior

## Abstract

***Introduction:*** Almost 2 million U.S. youth are estimated to live on the streets, in shelters, or in other types of temporary housing at some point each year. Both their age and living situations make them more likely to engage in high-risk behaviors, particularly during adolescence, a time of increased risk taking. Much of self-control appears related to the development of the prefrontal cortex, which is at a particularly crucial period of elaboration and refinement during adolescence and emerging adulthood. Executive processes like decision-making, inhibition, planning, and reasoning may be vulnerable to adversity experienced as a result of homelessness and related impoverishment during childhood and adolescence. No study to date, to our knowledge, has directly investigated differences in risk-taking by homeless youth as it relates to their developing executive control. ***Objective:*** Examine the relationship between the level of self-reported executive function (EF) and engagement in risk taking behaviors among a sample of shelter-living urban homeless youth. We predicted that homeless youth who have lower levels of self-reported EF would more readily engage in risky behaviors that could lead to negative outcomes. ***Participants:*** One hundred and forty-nine youths between 18 and 22 years of age were recruited from homeless agencies in Chicago. Of this study sample, 53% were female and 76% African American. ***Measures:*** All participants completed, as part of a broader neuropsychological assessment, the Behavior Rating Inventory of Executive Functioning-Adult Version (BRIEF-A), the National Youth Risk Behavior Survey (YRBS), and the Mini-International Neuropsychiatric Interview (MINI). ***Analyses:*** Groups were separated based on level of self-reported EF, with two groups identified: High self-reported EF fell >1 SD above the normative average, and low self-reported EF fell >1 SD below the normative average. All analyses utilized Chi-square and Mann-Whitney tests. ***Results and Conclusions:*** Analyses revealed a relationship between the level of self-reported EF and risk taking behaviors in this group of sheltered homeless urban youths. Those with lower self-reported executive functioning had higher rates of engagement in multiple substance-related risk taking behaviors. These findings are important because they are a first step towards identifying contributions to risk-taking behavior in urban homeless youths. Identifying potential factors like low self-reported EF better allows us to potentially intervene, thereby providing focused support to youths who are at higher risk for engaging in problematic behaviors.

## 1. Introduction

Youth homelessness in the United States is an important developmental issue. Studies have estimated that across a single year, there may be as many as 1.6–2 million youth living on the streets, in shelters, or in some other type of temporary housing [1,2,3,4]. Federal estimates suggest, based on point-in-time analysis, that 34% of the homeless population in the US is comprised of individuals under the age of 24, both individually or with family members, on any given night [5]. Although there are several different definitions proposed regarding what it means to be “homeless”, for the purposes of this investigation, we will parallel similar studies and define a homeless individual as someone without a fixed and consistent residence or whose residence is considered temporary or not designed for human habitation [2]. Notedly, homeless youth are a heterogeneous population but certain common challenges have been identified that precede their onset of homelessness. These include physical, psychological, or sexual abuse; family problems; economic hardships; mental illness; substance abuse or addiction; and aging out of the foster care system. These challenges represent significant life issues, which can contribute to an increased risk for homelessness and psychological burden [2,6,7,8].

A predominant focus in the current literature regarding youth homelessness is the tendency for these youth to engage in risky behaviors, such as unprotected sexual activity and substance abuse. It is important to understand, however, that adolescence is a time period of increased risk-taking regardless of one’s housing status [9,10,11,12,13,14]. This increased level of risk taking is believed to be principally a response to ongoing neurological changes, including a significant peak in brain maturation and development, particularly in the prefrontal cortex, that takes place during adolescence. The prefrontal cortex has been identified as the region largely responsible for managing executive functioning, a set of higher-order cognitive skills that includes processes such as decision-making, inhibition, reasoning, working memory, planning, and emotion and behavior regulation [2,5,9,10,11,12,13,14]. Studies of housed youth using magnetic resonance imaging (MRI) have shown that the brain continues to mature and develop until individuals are well into their twenties [2,15,16,17,18]; this highlights the protracted developmental period associated with executive skill development. More specifically, areas of myelinated white matter increase linearly with age, while areas of gray matter follow an inverted U-pattern of development. This linear increase in white matter is important because it allows for quicker and more efficient connections between different regions of the brain, supporting information processing. The inverted U-pattern of gray matter development represents a process known as synaptic pruning. This is significant because it supports an increase in the strength of frequently used brain circuits, and a decrease in those used less frequently. Until these processes of maturation and remodeling are complete, individuals are more prone to seek out and engage in impulsive actions, like risky behaviors, due to the fact that their executive functioning capacities are less developed and less successfully managed neurologically [2,15,17,18].

Although it has been shown that both homeless and housed adolescents engage in higher rates of risk taking and sensation seeking when compared to adults, these behaviors have been found to be more pronounced and longer lasting in their presentation in homeless youth, as compared to housed youths [2]. It has been suggested that this difference is a consequence of the impact of negative environmental factors, such as poverty and trauma, on the rate and completeness of brain development taking place during this crucial time period [2,19], leading to reduced executive control and poorer self-regulation. Specifically, impoverished environments (e.g., limited resources and available supports; increased likelihood of trauma; greater exposure to violence), such as those homeless youths often live within, impact the rate and completeness of myelination, contributing to smaller cortical size [19] and consequent inefficiencies in executive skill development. Lipina and Colombo have described the accumulated animal literature and now growing human literature that is supportive of influences of poverty and concurrent trauma and their associated challenges on both brain and behavioral development, including temperament, social relatedness, and cognitive functioning [19,20]; they have suggested that a greater hit is taken to maturing capacities for problem solving, strategy development and implementation, and flexibility in individuals experiencing substantial socioeconomic adversity.

Although a number of studies have looked at the differences in risk taking between homeless and housed individuals [1,9,10], there has been very little research investigating within-group differences in risk taking behavior that occur within the homeless youth population. While we cannot assume that all homeless youth engage in risky behaviors, those who do participate in risky activities often do so to differing degrees, and in relationship to differing internal and external demands. Given this variability, this study was designed to seek a better understanding of what factors differ among homeless youth that make some more likely to engage in high-risk behaviors versus their peers who do not, utilizing data obtained from a broader study of the impact of homelessness on cognitive and behavioral development. We hypothesized that differences in cognitive and executive functioning among our subset of homeless youth may contribute to their tendency to engage in risky behaviors; specifically, we hypothesized that shelter-living homeless youths with lower executive functioning profiles (based on self-report on a standardized questionnaire regarding daily executive functioning skill, the Behavior Rating Inventory of Executive Functioning, or BRIEF) would report higher levels of involvement in risk taking behaviors (e.g., condomless sex; substance abuse) when compared to their higher executive functioning counterparts. Our overall goal with regard to this study is to eventually be able to better identify homeless youths who are at greater risk for engaging in problematic behaviors, based on their executive functioning abilities. This study therefore serves as a first step in being able to identify differences in risk behavior in relationship to executive skill development. We believe that results from this work may provide a better path towards both support and education to help prevent them from potential self-endangerment and poorer developmental outcomes, and as well to better understand and promote areas of resilience in homeless youth.

## 2. Methods

### 2.1. Participants

One hundred and forty-nine participants were recruited from two homeless youth shelters in Chicago; the Teen Living Programs (TLP) and The Night Ministry (TNM). Interested youths were referred by TLP and TNM staff or were approached by study personnel on site and given details about the study. Those opting to participate, who met inclusion criteria including (1) individuals who lack a fixed, regular, and adequate nighttime residence; (2) were between 18 and 24 years of age at the time of assessment; (3) resided in Chicago; (4) spoke English as their primary language; and (5) had a measured IQ ≥ 65, were enrolled, consented, and evaluated. Six participants were removed from analyses due to missing information. Demographic information for participants can be found in Table 1.

### 2.2. Procedure

The study was approved by the University of Chicago Medicine and Biological Sciences Division Institutional Review Board (IRB). Review of the procedures underwent significant consideration by the IRB, given the sensitivity and specificity of the study aims and the population being assessed. Approval from the University’s IRB was based on assurances that our team made, in conjunction with the staff of the shelters, for the protection of the participants in this study. 

After informed consent was obtained, and following discussion with each youth regarding the sensitivity of the questions being asked and their ongoing right to maintain privacy and to minimize potential re-traumatization, each participant completed a psychosocial interview and was administered a full neurocognitive battery, over the course of two sessions. All testing was completed by trained research staff, who had a minimum of an undergraduate education and who achieved appropriate levels of administration skill consistent with the Department’s psychometrician staff. All interviews were conducted with the youth were administered by trained graduate students in clinical psychology or medicine, who were supervised by a licensed clinical psychologist and a board-certified child and adolescent psychiatrist, both of whom specialize in working with youth and families who have experienced trauma and adversity. Participants were provided with a $10 gift card as compensation for their time after the first session and a $20 gift card following completion of the second session.

For all participants in the homeless youth study, the first session, lasting approximately 1.5 h, included administration of the following measures: a detailed demographic interview administered by a trained research assistant that allowed for the collection of information addressing multiple areas of the participant’s early life, their upbringing, and their adolescence, and included questions regarding their experiences of homelessness, foster care, and education; the Adult Temperament Questionnaire (ATQ), a self-report model of temperament [21]; the Behavior Rating Inventory of Executive Function (BRIEF) [22], a well-standardized and normed self-report measure that assesses a range of everyday examples of executive functioning abilities, including inhibition, set-shifting, emotional control, task initiation, working memory, planning/organizing, self-organization of materials, and self-monitoring providing three composite scores, the Metacognition Index (MI), Behavior Rating Index (BRI), and Global Executive Composite (GEC) Index, which were examined with this study; the Mini International Neuropsychiatric Interview, Sixth edition (MINI), a semi-structured interview allowing for assessment of DSM-IV-TR based diagnoses and substance use disorders [23,24]; an adapted version of the 2011 Youth Risk Behavior Survey (YRBS), a standardized, self-report multiple-choice questionnaire that assesses recent and lifetime participation in common risky behaviors that has been used in previous research with homeless youth [25,26]; and lastly, the Wechsler Abbreviated Scale of Intelligence (WASI) [27], a measure of academic achievement which provides a full scale IQ score based on completion of four subtests addressing verbal and nonverbal problem solving.

The second session, lasting approximately 2.5 h, included administration of a number of neuropsychological tests, including the California Verbal Learning Test, Second Edition (CVLT-II) [28], an evaluation of multi-trial learning and long term recall of verbal information; the Delis-Kaplan Executive Functioning System (D-KEFS) [29], a well standardized objective assessment of executive functioning, including both verbal and nonverbal tasks; the Iowa Gambling Task (IGT) [30], a test of behavioral decision-making that addresses aspects of risk-taking and executive development; the Nelson-Denny Reading Test [31], a standardized and normed measure of vocabulary development, reading comprehension, and reading rate; the Drexel version of the Tower of London (TOL), an executive assessment measuring higher order problem solving [32]; the Weinberger Adjustment Inventory (WAI) [33], a self-report assessment of social-emotional adjustment in the context of external constraints; two subtests from the Wide Range Assessment of Memory and Learning, Second Edition (WRAML2) [34], assessing both immediate and delayed memory for verbal and nonverbal information; and the Wide Range Achievement Test, Fourth Edition (WRAT4) [35], a standardized and well-normed assessment of basic academic skills, including spelling, word reading, and basic calculations.

For this analysis and the questions posed with this study, we specifically focused on the demographic and semi-structured interview questions regarding current behavioral and adaptive functioning, and their relationship to profile responses to the BRIEF. The MINI was used to identify youths who met DSM-IV-TR criteria for alcohol dependence and abuse through a series of questions regarding their alcohol consumption [23]. The YBRS was used to identify the regularity and level of each study participant’s engagement in several common risky behaviors including experiences with alcohol, marijuana, other drugs, and their engagement in sex with multiple partners. The three core BRIEF composite scores where used to assess self-reported executive functioning. As mentioned above, these include the BRI, which measures the ability to modulate emotions via inhibitory control; the MI, which measures the ability to plan and sustain future oriented problem solving; and the GEC, which measures overall executive function [22].

### 2.3. Statistical Analyses

Norm-referenced clinically based determinants of high and low self-reported executive functioning (EF) were utilized and two groups were identified, based on the composite scores from the three factors of the BRIEF; the Global Executive Composite (GEC), Behavioral Regulation Index (BRI), and Metacognition Index (MI). Those who scored at least one full standard deviation below the normative population mean T-score of 50 were labeled as high functioning in regard to their demonstration of executive skill (e.g., those whose T-scores were at or below 40 on the BRIEF factors, utilizing a standard deviation of 10 points), and those who scored at least one standard deviation above were labeled as low functioning (e.g., those obtaining T-scores at or above 60 on the BRIEF factors, utilizing the normative mean of 50 and a standard deviation of 10 points). Chi-square and Mann-Whitney U tests were conducted to determine if self-reported executive functioning predicted engagement in risky behavior as assessed with the YRBS. In cases of dichotomous outcomes, such as MINI substance abuse and dependence, Chi-square tests were used. Because of the low expected cell counts in some Chi-square analyses, which generally assume expected cell counts above 5 (e.g., Bewick, Cheek, and Ball, 2004), Maximum Likelihood Ratio variants of the Chi-square test were utilized and reported in analyses in which this assumption was violated. For ordinal outcomes in which normality of scores and homogeneity of variance cannot be assumed, such as YRBS items, Mann-Whitney *U* tests were utilized for comparisons [36].

## 3. Results

### 3.1. Participant Executive Function

As discussed above, participants were categorized as having either high or low self-reported EF based on their three composite scores from the BRIEF, GEC, BRI, and MI. A comprehensive summary of results from these cognitive measures can be seen in Table 2.

### 3.2. Alcohol Abuse and Dependence

In order to determine if differences in self-reported alcohol abuse existed between low and high self-reported EF youths, Chi-square tests of independence were conducted. We found that the youths who had low self-reported EF had significantly higher rates of alcohol abuse, and this was true across all factors of the BRIEF, including the GEC (*p* = 0.002), BRI (*p* = 0.003), and MI (*p* = 0.016) factors. A comprehensive summary of the findings regarding alcohol abuse can be seen in Table 3. Effect sizes for all three Chi-square tests were in the moderate to large range (between 0.34 and 0.43).

Next, Chi-square tests of independence were conducted to determine if a difference in alcohol dependence classification existed between low and high self-reported EF youths. We found that the youths who had low self-reported EF had significantly higher rates of likely alcohol dependence based on DSM-IV criteria from the MINI, and this was true when examining the GEC (*p* < 0.001), BRI (*p* = 0.008), and MI (*p* = 0.001) factors of the BRIEF. A comprehensive summary of the findings regarding alcohol dependence can be seen in Table 4. Effect sizes for all comparisons were also in the moderate to large range (between 0.36 and 0.43).

### 3.3. Additional Alcohol Findings

In order to assess differences in alcohol consumption patterns, participants were asked on the YRBS to indicate on how many consecutive days they had five or more alcoholic drinks, in the past 30 days. The YRBS scale ordinally categorizes the number of consecutive days into seven levels representing: 0, 1, 2, 3–5, 6–9, 10–19, and ≥20 days. Using Mann-Whitney tests, we found differences in alcohol consumption patterns between low and high self-reported EF individuals. For the BRI, the low self-reported EF group had a higher amount of consecutive days drinking five or more drinks (M = 1.00, SD = 1.59) as compared to the high self-reported EF group (M = 0.09, SD = 0.43), *U* = 209, *p* = 0.012, *r* = 0.36. Examining the overall GEC, the low EF participants shared having a higher number consecutive days drinking five or more drinks (M = 0.77, SD = 1.394) as compared to the high self-reported EF group (M = 0.11, SD = 0.577), *U* = 269.5, *p* = 0.02, *r* = 0.32, and this was also true for alcohol consumption pattern differences when defining EF based on the MI score (*U* = 220.5, *p* = 0.014, *r* = 0.35).

### 3.4. Drug Use and Sexual Behavior Findings

In order to assess differences in broader drug use between high and low EF youths, participants were asked on the YRBS to indicate how many times they had used marijuana in the past 30 days. The YRBS scale ordinally categorizes the number of times into six levels representing: 0, 1–2, 3–9, 10–19, 20–39, and ≥40 times. Using Mann-Whitney tests, we found that individuals with low self-reported EF as defined by the BRI, used marijuana more frequently during the indicated time period (M = 1.44, SD = 1.89) as compared to the high self-reported EF group (M = 0.36, SD = 0.95), *U* = 189.5, *p* = 0.012, *r* = 0.36. We did not find any significant differences when examining other drug use; of note, this may be the result of floor effects, as other drugs were much less commonly acknowledged being used by the relevant cohorts. We also did not find significant differences in marijuana use when comparing high and low EF groups as defined by the GEC (*U* = 280, *p* = 0.134) or MI (*U* = 220, *p* = 0.062).

Next, in order to assess differences in sexual practices between high and low self-reported EF youths, participants were asked on the YRBS how many lifetime sexual partners they have had. The YRBS scale ordinally categorizes the number of partners into seven levels representing: 0, 1, 2, 3, 4, 5, and ≥6 partners. Using Mann-Whitney tests, we found that individuals with low self-reported EF on the BRI had a higher number of lifetime sexual partners (M = 4.70, SD = 1.94) as compared to the high self-reported EF group (M = 3.64, SD = 1.87), *U* = 194, *p* = 0.029, *r* = 0.31. This comparison was also significant when defining EF by GEC score (*U* = 242, *p* = 0.043, *r* = 0.28), but not MI (*U* = 240.5, *p* = 0.228). Lastly, we examined whether differences existed in drug use in conjunction with sex between the high and low self-reported EF individuals, given the relationships that have been found in other studies regarding the use of drugs in conjunction with sex and the likelihood of engaging in risky sexual activities, such as condomless sex. Results from a Chi-square test of independence showed that those with low self-reported EF on the BRI had a higher proportion of drug use (19.2%) in conjunction with their last sexual encounter as compared to the high self-reported EF group (0%), χ^2^ (1, *N* = 46) = 6.17, *p* = 0.013, V = 0.306. Additionally, those with low self-reported EF on the GEC had a higher proportion of drug use (12.0%) in conjunction with their last sexual encounter as compared to the high self-reported EF group (0%), χ^2^ (1, *N* = 50) = 4.35, *p* = 0.037, V = 0.253. However, this comparison was not significant when defining EF by the MI (χ^2^ = 3.25, *p* = 0.072). 

## 4. Discussion

As anticipated, we found that there were significant differences in risk taking behavior and substance use between high and low self-reported EF participants. Specifically, we found that shelter-residing homeless youth with reported low EF (e.g., T-scores above 60 on the BRIEF) admitted to engaging in behaviors consistent with classification as alcohol abuse and endorsed symptoms consistent with alcohol dependence on the MINI. This remained true across all of the BRIEF composite scores, highlighting a relationship with low levels of behavior regulation and metacognition. Additionally, we found that those classified as low self-reported EF on the BRI reported drinking five or more alcoholic beverages across multiple consecutive days in comparison with their high self-reported EF (e.g., T-scores below 40) counterparts. Lastly, we found that those with low self-reported EF, as measured on the BRI, smoked marijuana more frequently within a specified time period (30 days, on the YRBS), and were more likely to endorse having used drugs during their last sexual encounter. These individuals also had a higher number of lifetime sexual partners when compared with their high self-reported EF homeless peers. Each of these findings is suggestive of a strong link between low self-reported EF and higher engagement in risk behavior, specifically the use of substances more regularly, and at a greater level than peers who are more admittedly executive. Additionally, a consideration is raised regarding the reasons for using substances more readily; those youth using at a higher level endorsed having much lower capacity for behavioral inhibition skills, including impulse control and the capacity to regulate emotional experience. This supports previous findings in the literature regarding the association of low EF and greater risk behavior, and highlights the need for further investigation into patterns of use and reasons for risk behavior choices among these homeless adolescents and emerging adults [37,38,39]. Considerations for rates of use and reasons for using during riskier behavioral choices may reflect efforts at self-regulation through substances, or cognitive deficiencies regarding decision-making, or an interrelationship between these possible responses within a broader contextual milieu of poverty and trauma.

There are several limitations noted with this study. First, our sample is comprised of shelter-dwelling homeless youth that opted to participate. It is possible that this group is higher functioning overall, in comparison with street-dwelling homeless peers. This has the potential to influence scores regarding the overall aspects of executive and behavioral functioning. Nonetheless, examination of our sample in regard to both intellectual functioning and academic skill development indicated that they are predominantly within the average range, with their scores falling approximately one standard deviation below the mean across all of the cognitive indices (i.e., the WASI, FSIQ, and WRAT-4 scores). Second, our sample was 75% African American, highlighting an overrepresentation of one racial group and their particular set of possible challenges in regard to being homeless. This may limit the generalizability of our findings to other homeless youth populations that are broader in their cultural and racial composition. Third, we recruited participants from two shelters within a single US city; although most likely representative of the homeless youth population of Chicago [40], this sample is limited in generalizability to different settings across the country, where homeless youth may face different challenges and be more or less likely to be exposed to particular risky behaviors and environmental demands. It is noted however that the percentage of homeless youth of color in Chicago is comparable to those in other major US cities [41]. Lastly, it is not possible to identify whether the deficits in EF are the cause of poor decision-making regarding risk behavior, or are a result of the engagement in risk behaviors, perhaps as a survival means, or due to some other factor not identified that represents a range of potential contextual concerns, such as poverty, low educational opportunity, or availability of substances. We believe that future investigations should attempt to address these limitations more directly, by engaging and assessing a broader sample of homeless youth, both in terms of their living setting and their location nationally. By expanding similar studies to other cities, we believe that this will diversify the sample both culturally and racially, and increase our understanding of the impact of homelessness and its challenges on youth development more broadly. 

Ultimately, this study provides a foundation for better identifying which factors that characterize homeless youth contribute to their engagement in significant patterns of risk-taking behavior. It appears that the development of specific patterns of executive functioning may well play an important role in understanding vulnerabilities and risk, as well as guide our understanding of resilience in this population, and how to best engage and promote EF capacities that underlie more effective and adaptive decision-making. With this information, it is believed that clinicians and counselors working with homeless youths will be able to both better identify those specific youth who are at a higher risk, in order to guide their participation in support programs and resources that will improve their outcomes, for both those that currently exist and ones that may be developed from the findings of ongoing research. Additionally, understanding the profile of risk neurobehaviorally requires further consideration of those youth who more effectively address the challenges homelessness presents, through their own resiliency as well as their capacity to take advantage of available community and social service resources. 

## 5. Conclusions

Homeless urban youth are a significantly vulnerable population, in response to their frequent histories of trauma and adversity, lack of economic and housing resources, challenges with consistent educational opportunity, and associated neurodevelopmental risk. Results from this study examining the relationship between developing EF and a history of homelessness, from a large community recruited sample of shelter-dwelling predominantly African-American urban youth, highlight that those youth with reduced profiles of current EF skill (assessed with the BRIEF) are more likely to engage in impulsive and riskier behaviors (including substance abuse and increased sexual partners), and meet criteria for substance abuse diagnoses. These negative behavioral profiles in low EF youth contribute to greater potential developmental morbidity and associated reduced opportunities for social and adaptive success. Recognizing that adolescence and emerging adulthood are particular times of increased risk, secondary to ongoing elaboration and refinement of the neural networks that support EF, understanding the relationship between homelessness and EF, and the broader range of contributing challenges that impact these youths’ experience [42] highlight an area ripe for increased research and clinical emphasis. Further work remains, to provide better knowledge that can guide clinical intervention and policy development. 

## Figures and Tables

**Table 1:** Participant demographics (full sample).

Demographic Variable	Value
Sample Size (N)	143
Mean age in years (SD)	19.28 (0.95)
Age range in years	18–22
Mean age of first homeless episode, in years (SD)	16.22 (3.78)
Age range of first homeless episode, in years	1–22
Mean number of homeless episodes (SD)	1.63 (1.2)
Range in number of homeless episodes	1–10
Mean longest episode of homelessness in months (SD)	12.83 (19.12)
Range in months of longest homelessness episodes	0.25–180
*Gender N* (%)	
Female	75 (51.7%)
Male	68 (46.9%)
*Race, percentile*	
African American	75.2%
Caucasian	4.1%
Multiracial	8.3%
Latino/Hispanic	5.5%
Other	5.5%

**Table 2:** Breakdown of participant self-reported executive function.

Measure of Executive Function	Total Sample (*n* = 143) M (SD)	High EF (N*)	Low EF (N*)
Behavioral Regulation Index	54 (12)	22	27
Metacognition Index	52 (11)	26	23
Global Executive Composite	53 (12)	27	26

SD = Standard Deviation, EF = Executive Function; N* = number of subjects falling ± 1 SD above (low) or below (high) mean.

**Table 3:** Chi-square results for relationship between self-reported EF and alcohol abuse proportions.

Measure of Executive Function	Alcohol Abuse	High EF	Low EF	X^2^	Cramer’s V
Behavioral Regulation Index	Yes	2 (9.1%)	13 (48.1%)	8.71 *	0.42
	No	20 (90.9%)	14 (51.9%)		
Metacognition Index	Yes	2 (7.7%)	8 (34.8%)	5.77 *	0.34
	No	24 (92.3%)	15 (65.2%)		
Global Executive Composite	Yes	1 (3.7%)	10 (38.5%)	9.73 *	0.43
	No	26 (96.3%)	16 (61.5)		

Note: * *p* = ≤ 0.05. EF = Executive Function.

**Table 4:** Chi-square results for relationship between self-reported EF and alcohol dependence proportions.

Measure of Executive Function	Alcohol Dependence	High EF	Low EF	X^2^	Cramer’s V
Behavioral Regulation Index	Yes	1 (4.5%)	9 (33.3%)	7.08 *	0.36
	No	21 (95.5%)	18 (66.7%)		
Metacognition Index	Yes	0 (0%)	7 (30.4%)	11.92 *	0.43
	No	26 (100%)	16 (69.6%)		
Global Executive Composite	Yes	0 (0%)	8 (30.8%)	12.88 **	0.43
	No	27 (100%)	18 (69.2%)		

Note: * *p* ≤ 0.05. ** *p* ≤ 0.001. EF = Executive Function.

## References

[1] Castro A.L., Gustafson E.L., Ford A.E., Edidin J.P., Smith D.L., Hunter S.J., Karnik N.S. (2014). Psychiatric disorders, high-risk behaviors, and chronicity of episodes among predominantly African American homeless Chicago youth. J. Health Care Poor Underserv..

[2] Edidin J.P., Ganim G., Hunter S.J., Karnik N.S. (2012). The mental and physical health of homeless youth: A literature review. Child Psychiatry Hum. Dev..

[3] Tompsett C.J., Domoff S.E., Toro P.A. (2013). Peer substance use and homelessness predicting substance abuse from adolescence through early adulthood. Am. J. Commun. Psychol..

[4] Yu M., North C.S., Lavesser P.D., Osborne V.A., Spitznagel E.L.A. (2008). Comparison study of psychiatric and behavior disorders and cognitive ability among homeless and housed children. Commun. Ment. Health J..

[5] Henry M., Shivji A., de Sousa T., Cohen R., Khadduri J., Culhane D.P. (2015). The 2015 Annual Homelessness Assessment report (AHAR) to Congress, Part 1: Point-in-Time Estimates of Homelessness. https://www.hudexchange.info/resources/documents/2015-AHAR-Part-1.pdf.

[6] Burra T.A., Stergiopoulos V., Rourke S.B.A. (2009). Systematic review of cognitive deficits in homeless adults: Implications for service delivery. Can. J. Psychiatry.

[7] Fowler P.J., Toro P.A., Miles B.W. (2009). Pathways to and from homelessness and associated psychosocial outcomes among adolescents leaving the foster care system. Am. J. Public Health.

[8] Shelton K.H., Taylor P.J., Bonner A., Van Den Bree M. (2009). Risk factors for homelessness: Evidence from a population-based study. Psychiatr. Serv..

[9] Arnett J. (1992). Reckless behavior in adolescence: A developmental perspective. Dev. Rev..

[10] Bernheim A., Halfon O., Boutrel B. (2013). Controversies about the enhanced vulnerability of the adolescent brain to develop addiction. Front. Pharmacol..

[11] Crone E.A. (2009). Executive functions in adolescence: Inferences from brain and behavior. Dev. Sci..

[12] Johnson S.B., Blum R.W., Giedd J.N. (2009). Adolescent maturity and the brain: The promise and pitfalls of neuroscience research in adolescent health policy. J. Adolesc. Health.

[13] Romer D. (2010). Adolescent risk taking, impulsivity, and brain development: Implications for prevention. Dev. Psychobiol..

[14] Steinberg L. (2004). Risk taking in adolescence: What changes, and why?. Ann. N. Y. Acad. Sci..

[15] Blakemore S.J., Suparna C. (2006). Development of the adolescent brain: Implications for executive function and social cognition. J. Child Psychol. Psychiatry Allied Discip..

[16] Lenroot R., Giedd J.N. (2006). Brain development in children and adolescents: Insights from anatomical magnetic resonance imaging. Neurosci. Biobehav. Rev..

[17] Giedd J.N., Rapoport J.L. (2010). Structural MRI of pediatric brain development: What have we learned and where are we going?. Neuron.

[18] Giedd J.N., Blumenthal J., Jeffries N.O., Castellanos F.X., Liu H., Zijdenbos A., Paus T., Evans A.C., Rapoport J.L. (1999). Brain development during childhood and adolescence: A longitudinal MRI study. Nat. Neurosci..

[19] Lipina S.J., Posner M.I. (2012). The impact of poverty on the development of brain networks. Front. Hum. Neurosci..

[20] Lipina S.J., Colombo J.A. (2009). Poverty and Brain Development during Childhood: An Approach from Cognitive Psychology and Neuroscience.

[21] Evans D.E., Rothbart M.K. (2007). Development of a model for adult temperament. J. Res. Personal..

[22] Gioia G.A., Isquith P.K., Guy S.C., Kenworthy L. (2000). Behavior Rating Inventory of Executive Function (BRIEF).

[23] American Psychiatric Association (2000). DSM-IV-TR: Diagnostic and Statistical Manual of Mental Disorders, Text Revision.

[24] Sheehan D.V., Lecrubier Y., Sheehan K.H. (1998). The mini-international neuropsychiatric interview (M.I.N.I.): The development and validation of a structured diagnostic psychiatric interview for DSM-IV and ICD-10. J. Clin. Psychiatry.

[25] Centers for Disease Control and Prevention (CDC) (2011). 2011 Youth Risk Behavior Survey. https://www.cdc.gov/healthyyouth/data/yrbs/questionnaires.htm.

[26] Rice E., Fulginiti A., Winetrobe H., Montoya J., Plant A., Kordic T. (2012). Sexuality and homelessness in Los Angeles public schools. Am. J. Public Health.

[27] Wechsler D. (1999). Wechsler Abbreviated Scale of Intelligence (WASI).

[28] Delis D.C., Kramer J.H., Kaplan E., Ober B.A. (2000). California Verbal Learning Test: Second Edition (CVLT-II).

[29] Delis D.C., Kaplan E., Kramer J.H. (2001). Delis Kaplan Executive Function System (D-KEFS).

[30] Bechara A. (2007). Iowa Gambling Task (IGT).

[31] Brown J.A., Fishco V.V., Hanna G. (1993). Nelson–Denny Reading Test: Manual for Scoring and Interpretation, Forms G & H.

[32] Culbertson W.C., Zilmer E.A. (2005). Tower of London Drexel University: Second Edition (TOLDX2).

[33] Weinberger D.A., Feldman S.S., Ford M.E., Chastain R.L. (1987). Construct Validation of the Weinberger Adjustment Inventory.

[34] Sheslow D., Adams W. (2003). Wide Range Assessment of Memory and Learning Second Edition (WRAML2).

[35] Wilkinson G.S., Robertson G.J. (2006). Wide Range Achievement Test 4 Professional Manual.

[36] Bewick V., Cheek L., Ball J. (2003). Statistics review 8: Qualitative data–tests of association. Crit. Care.

[37] Ursache A., Raver C.C. (2015). Iowa Gambling Task performance and executive function predict low-income urban preadolescents’ risky behaviors. Personal. Individ. Differ..

[38] Steinberg L. (2008). A social neuroscience perspective on adolescent risk-taking. Dev. Rev..

[39] Nixon S.J. (2013). Executive functioning among young people in relation to alcohol use. Curr. Opin. Psychiatry.

[40] Voorhees Center for Neighborhood and Community Improvement, University of Illinois at Chicago (2016). City of Chicago 2016 Homeless Point-in-Time Count & Survey Report.

[41] National Alliance to End Homelessness Home Page. http://www.endhomelessness.org/.

[42] Fry C.E., Langley K., Shelton K.H. (2017). A systematic review of cognitive functioning among young people who have experienced homelessness, foster care, or poverty. Child Neuropsychol..

